# Degradation Prediction Model Based on a Neural Network with Dynamic Windows

**DOI:** 10.3390/s150306996

**Published:** 2015-03-23

**Authors:** Xinghui Zhang, Lei Xiao, Jianshe Kang

**Affiliations:** 1 Mechanical Engineering College, Shijiazhuang 050003, China; E-Mails: dynamicbnt@aim.com (X.Z.); jskang201206@aim.com (J.K.); 2 The State Key Lab of Mechanical Transmission, Chongqing University, Chongqing 400030, China; 3 Department of Industrial and Systems Engineering, Rutgers University, Piscataway, NJ 08854, USA

**Keywords:** degradation tracking, remaining useful life, neural network, dynamic window

## Abstract

Tracking degradation of mechanical components is very critical for effective maintenance decision making. Remaining useful life (RUL) estimation is a widely used form of degradation prediction. RUL prediction methods when enough run-to-failure condition monitoring data can be used have been fully researched, but for some high reliability components, it is very difficult to collect run-to-failure condition monitoring data, *i.e.*, from normal to failure. Only a certain number of condition indicators in certain period can be used to estimate RUL. In addition, some existing prediction methods have problems which block RUL estimation due to poor extrapolability. The predicted value converges to a certain constant or fluctuates in certain range. Moreover, the fluctuant condition features also have bad effects on prediction. In order to solve these dilemmas, this paper proposes a RUL prediction model based on neural network with dynamic windows. This model mainly consists of three steps: window size determination by increasing rate, change point detection and rolling prediction. The proposed method has two dominant strengths. One is that the proposed approach does not need to assume the degradation trajectory is subject to a certain distribution. The other is it can adapt to variation of degradation indicators which greatly benefits RUL prediction. Finally, the performance of the proposed RUL prediction model is validated by real field data and simulation data.

## 1. Introduction

Mechanical faults can lead to whole system failure and great economic losses. Especially for key assets like helicopters or wind turbines, the possible consequences of component failure are serious. In order to plan the maintenance actions and prevent potential failure, many researchers have paid great attention to tracking the degradation of mechanical components. Usually, remaining useful life (RUL) estimation is a widely used form of degradation prediction which can help make maintenance decisions [1].

Most previous works use a sufficient number of failure data to train prediction models. It is widely accepted that the more failure histories are used to train the models, the more accurate results can be achieved. In industry, besides the failure history data, there is suspension data. In order to overcome the problem of a limited amount of historic data, Tian *et al*. [2] proposed a neural network prediction method which can utilize both failure and suspension histories data. Then, for alleviating the fluctuation of degradation indicators, a function generalized from the Weibull failure rate function was used to fit condition indicators [3]. Recently, Lu *et al*. [4] developed an effective RUL prediction method based on a feed-forward neural network. In the paper, only truncated histories were used to train the model. Zhou *et al*. [5] proposed a state space RUL prediction model without linear and Gaussian assumptions. In the model, an efficient Monte Carlo-based algorithm was developed to estimate the parameters. Zhang *et al*. [6] developed a Bayesian networks-based degradation model. It can achieve accurate RUL values even when the degradation indicator fluctuates over a great range. Besides, maintenance decisions can be made efficiently according to the degradation state identification. Dong and He [7] constructed an integrated diagnostics and prognostics framework based on a hidden semi-Markov model (HSMM). Similarly, the extended HSMM is widely applied to RUL prediction [8,9,10]. Moghaddass and Zuo [11] proposed a new integrated diagnostics and prognostics framework based on non-homogeneous continuous time HSMM. Si *et al*. [12] proposed a Wiener process-based degradation model in which a recursive filter algorithm was used to update parameters. Then, Wang *et al*. [13] developed a new degradation prediction framework in which an additive Wiener process model contained both linear and nonlinear parts. Wang *et al*. [14] developed an adaptive RUL prediction method based on a generalized Wiener process. The Wiener process also was used to predict the RUL of 2008 PHM competition data [15]. Lately, an adaptive and nonlinear prognostic model to estimate RUL using a system history of the observed data to date was presented [16]. Ye *et al*. [17] developed a semi-parametric inference method of a simple Gamma-process model and a random-effect variant. This enabled the Gamma process-based degradation model results to be close to the practice. Recently, Ye and Chen [18] systematically investigated the characteristics of an inverse Gaussian process as a degradation model. Then, based on this work, Peng *et al*. [19] studied the inverse Gaussian model from a Bayesian perspective. Recently, Ye and Xie [20] systematically reviewed degradation models, especially for stochastic processes models. However, the efficiency of stochastic processes-based degradation models depends on the proper estimation of some prior distributions and parameters of the model. In addition, a number of failure or suspension histories are needed. To the best of our knowledge, prediction models adaptive to a limited number of condition monitoring data have not been fully researched yet.

Tran *et al*. [21] proposed a one-step-ahead prediction method based on regression trees. Similarly, Tian and Zuo [22] used an extended recurrent neural network to do one-step-ahead prediction for gear degradation. However, one-step-ahead method has drawbacks. It cannot directly be used to predict RUL. Tran *et al*. [23] developed a multi-step ahead prediction method using regression trees and neuro-fuzzy systems. Similarly, this method cannot solve the RUL prediction problem. Chen *et al*. [24] developed an integrated adaptive neuro-fuzzy and high-order particle filtering approach to predict the evolution of fault indicators and estimate the probability density function of RUL. This method overcomes the limitation that the current state of system only depends on the previous state. It can improve the prediction accuracy. He *et al*. [25] developed an integrated RUL prediction method using particle filtering, then applied it to gear life prediction. Ma and He [26] improved the trending of the fault features through combining vibration analysis with grease debris analysis based on a particle filtering framework. Recently, Yoon and He [27] developed a new prognostic estimation technique. Different from previous particle filtering-based estimation methods, the proposed model was a hybrid of the unscented Kalman filter and particle filtering. Maio *et al*. [28] developed a degradation trend prediction method based on a relevance vector machine and exponential regression. It can deal with degradation indicators with low signal-to-noise ratios caused by different working conditions and failure severity. However, existing degradation trend prediction methods also have the following problems: (1) When the steps in the long-term rolling prediction exceed a certain value, the predicted value will be same or will fluctuate in a certain range. This makes RUL prediction unavailable; (2) Degradation indicators extracted from vibration signals usually display large fluctuations and the degradation paths vary with the different rates at each phase. This results in large variance of the estimated RUL.

In order to solve the problems mentioned above, this paper proposes a new adaptive degradation prediction method based on neural network combined with dynamic windows. The main contributions of this paper can be concluded as follows: (1) A new adaptive prediction model which adapts to various degradation paths is developed. It achieves this goal by adjusting the training window adaptively according to real data; (2) A dynamic window adjusting method based on increasing rate is proposed. It can realize RUL predictions using only a limited number of condition monitoring data with high prediction accuracy; (3) The change point detection method and window adjusting method of this case is proposed. This method can enable determination of predicted values more precisely by using the indicators after change point.

The rest of this paper is organized as follows: Section 2 explains the drawbacks of traditional rolling prediction based on neural networks. In order to overcome these drawbacks, a new prediction method with dynamic windows is proposed in Section 3. In Section 4, two simulated degradation indicators of two different components are used to validate the proposed method. The proposed method is validated by two real-field datasets in Section 5 and Section 6, respectively. Finally, Section 7 concludes the whole paper and presents prospects for future research.

## 2. Traditional Neural Network Based Prediction Model

### 2.1. Problem Description

**Figure 1:**
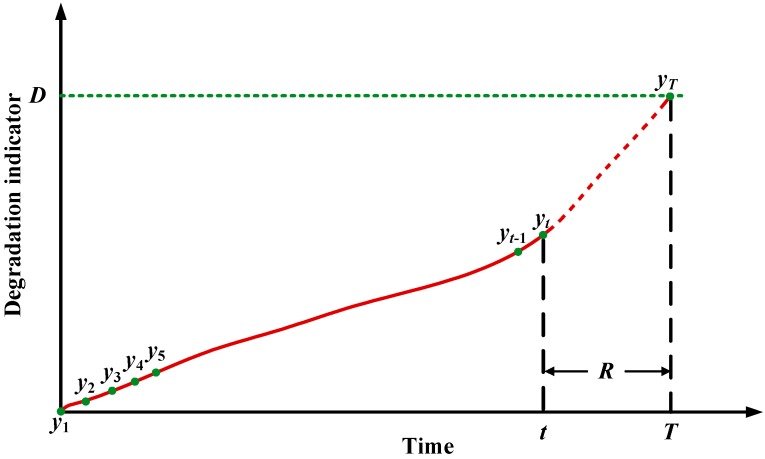
Illustration of degradation process and RUL prediction.

RUL is usually selected as the index for degradation prediction and is used to schedule maintenance actions. For different degradation indicators, there are different forms of RUL. In this paper, degradation indicators extracted from vibration signals is used to predict RUL. Suppose the inspection interval is equal. The degradation indicators can be denoted as ***Y*** = [*y*_1_, *y*_2_, … *y_t_*_-1_, *y_t_*, …. *y_T_*]. *T* is the failure time or the time of degradation indicator hitting threshold. Because indicators used for training and prediction should be normalized before being input into the neural network, therefore, all the indicator vectors used in this paper are supposed to be normalized vectors. If it is predicted at time *t*, RUL will be *T*-*t*. This is illustrated in Figure 1. The dashed line is the predicted degradation indicators. *D* denotes the failure threshold.

### 2.2. Rolling Prediction Based on a Neural Network

For some components like gearboxes in wind turbines or helicopters, collecting condition monitoring data from normal to failure is very difficult. Especially for new types of gearbox which have not been used, no failure history can be used for RUL prediction. Only the collected condition monitoring data from in use performance can be used. Traditionally, two kinds of rolling prediction methods are used. The first is to predict one future indicator value according to a series of former indicators. For example, degradation indicators [*y*_1_, *y*_2_, *y*_3_, *y*_4_, *y*_5_] are used to predict the indicator *y*_6_. Then, [*y*_2_, *y*_3_, *y*_4_, *y*_5_, *y*_6_] is used to predict the value of *y*_7_. This process is repeated until the predicted indicator exceeds some predefined failure threshold. The second way is to predict a series of future indicators according to a series of former indicators. For example, degradation indicators [*y*_1_, *y*_2_, *y*_3_, *y*_4_, *y*_5_] are used to predict the indicators [*y*_6_, *y*_7_, *y*_8_, *y*_9_, *y*_10_]. Then, [*y*_6_, *y*_7_, *y*_8_, *y*_9_, *y*_10_] is used to predict the value of [*y*_11_, *y*_12_, *y*_13_, *y*_14_, *y*_15_]. Similarly, this prediction process will stop once the predicted indicator exceeds the predefined failure threshold.

**Figure 2:**
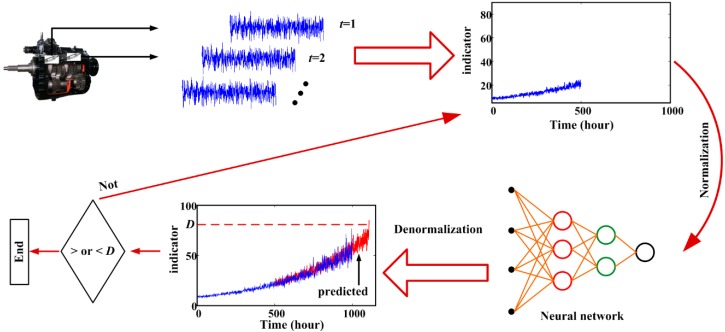
RUL estimation using rolling prediction.

A back propagation neural network (BPNN) is used to conduct rolling prediction. Roughly speaking, it is a black box which obtains output according to the input with regardless of the mechanism between input and output. It constructs the relationship between input and output by adjusting the connection weights among neurons. The rolling prediction framework based on a neural network is illustrated in Figure 2.

### 2.3. The Disadvantages of Neural Network-Based Prediction

Though neural networks have many advantages like good fitting of time series data, their extrapolability is poor, especially for long-term prediction. The main reason is resulted from small training samples and the range of activation function of the neurons, so the output converges on a certain value or fluctuates in a small range. In order to illustrate these phenomena, a simple example is given. Degradation indicators are simulated using the degradation model presented in [29,30]:

(1) $$s\left(t\right)=\varphi +\theta \text{exp}\left(\beta t+\varepsilon \left(t\right)-\frac{\sigma^{2}t}{2}\right)$$

where *s*(*t*) denotes a continuous degradation indicator extracted from vibration signals, *φ* is a constant, *θ* is a lognormal random variable, ln*θ* has mean *μ*_0_ and variance (*σ*_0_)^2^. *β* is a normal random variable with mean *μ*_1_ and variance (*σ*_1_)^2^. *ε*(*t*) is a centered Brownian motion with mean 0 and variance *σ*^2^*t*.

**Figure 3:**
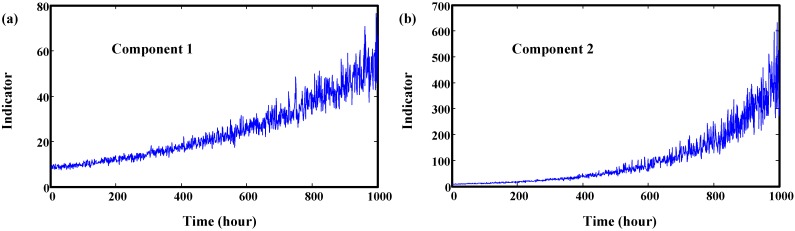
Simulated degradation indicators of two components. (**a**) Component 1; (**b**) Component 2.

In this simulation, *φ* is equal to 1. The mean and standard deviation of lognormal distribution of *θ* are 2 and 0.02. The mean and standard deviation of *β* are 1 and 0.05. *σ* of Brownian motion is equal to 0.05. The simulated indicators of two components are illustrated in Figure 3.

Figure 3a shows that degradation indicators generally increase from the beginning of the working time of Component 1. However, the degradation indicator of Component 2 does not increase in the initial state. In engineering applications, components of different type will have different degradation processes. Even components from the same type may have different degradation processes, so two different degradation processes of different component were simulated. Suppose the time interval between two successive inspections is 1 h.

**Figure 4:**
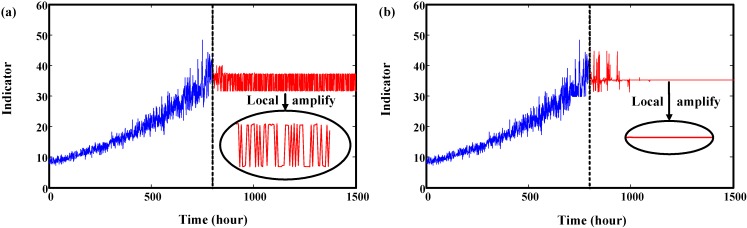
Results of rolling prediction. (**a**) Results fluctuate in a range; (**b**) Results keep the same value.

Let us take the simulation data of Component 1 as example. The multi-step ahead prediction mentioned in Section 2.2 is used to predict RUL. Indicators before prediction time *t* are divided into two groups. If *t* = 100 h, each group contains 50 indicators, respectively, so the first 50 indicators [*y*_1_, *y*_2_, …*y*_50_] are used as input data. The second 50 indicators [*y*_51_, *y*_52_, … *y*_100_] are used as output data. In other words, the first 50 indicators are used to predict the next 50 indicators, which is actually a 50-step ahead prediction. For the neural network, the number of neurons in hidden layer is selected 10. Learning rate is 0.05. Maximum iteration epoch is 1000. Error goal is 1e−10. The activation functions in the hidden layer and output layer are ‘logsig’ and ‘purelin’, respectively. Weights are adjusted based on gradient descent method. In the rest of this paper, all the neural network parameters are the same. Figure 4 shows the two results achieved by rolling prediction.

Figure 4a shows that the predicted values fluctuate within a certain range. However, sometimes the predicted values converge to a certain value as shown in Figure 4b. In both cases, the predicted values cannot exceed the predefined threshold. Therefore, the traditional BPNN method cannot be used to predict RUL.

## 3. Proposed Neural Network-Based Prediction Model

Most rotating machinery undergoes a gradual degradation process. It is a long period during which indicators show only small range variations. In that case, we assume that there is no need to predict RUL in that period. Prediction should be conducted after incipient fault is found. The incipient faults of these rotating parts can be found through signal processing analysis. Thus, the prediction time *t* can be determined. The degradation indicators before time *t* are used to train neural network. The newly developed prediction method mainly contains three parts: (1) degradation indicator extraction; (2) window size determination and (3) rolling prediction. Because indicator extraction is not the main scope of this paper, the other two parts will be explained. All the cases suppose that degradation indicators are available.

### 3.1. Window Size Determination

**Figure 5:**
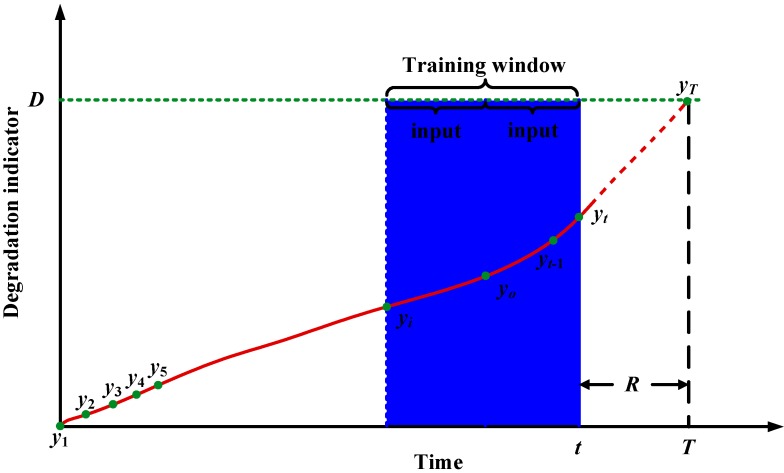
Illustration of the training window.

A neural network predicts the degradation indicator trends mainly depending on the corresponding training data trend. If the training data does not have an obvious trend, it is difficult to predict future degradation indicators using a neural network, so training data selection is very important for RUL prediction. We name the coordinate range of the training data shown in Figure 5 as the training window.

Window size is a dynamic value for each prediction time. That means the window size should be adjusted before the prediction. The window size of training data can be determined through two steps. First, an initial window size is determined randomly. Then, the window size is gradually adjusted based on the increasing rate of the window. Second, detecting whether there is change point in the training window or not. If there is change point, indicators after the point are used to train the prediction model, rather than the indicators in window determined by the first step.

#### 3.1.1. Window Size Determination Based on Increasing Rate

**Figure 6:**
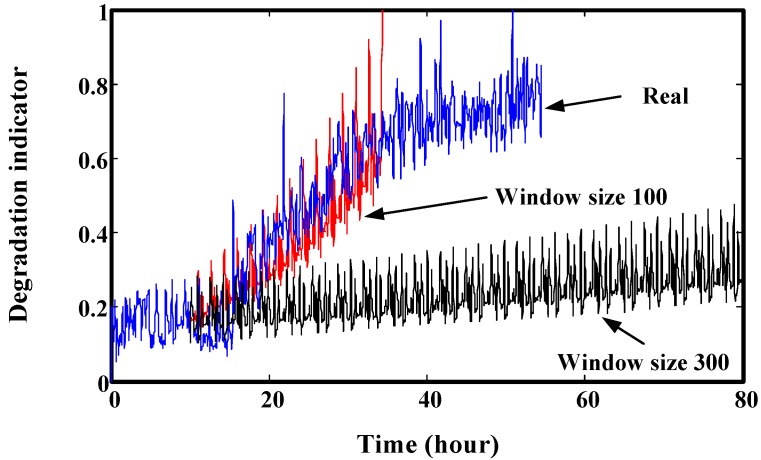
Prediction results comparison using different window sizes (start at 10 h).

**Figure 7:**
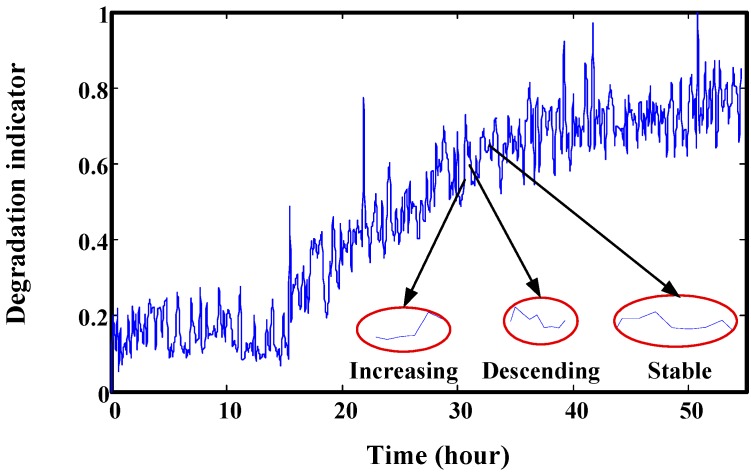
Three typical results from small windows: descending, increasing and stable.

**Figure 8:**
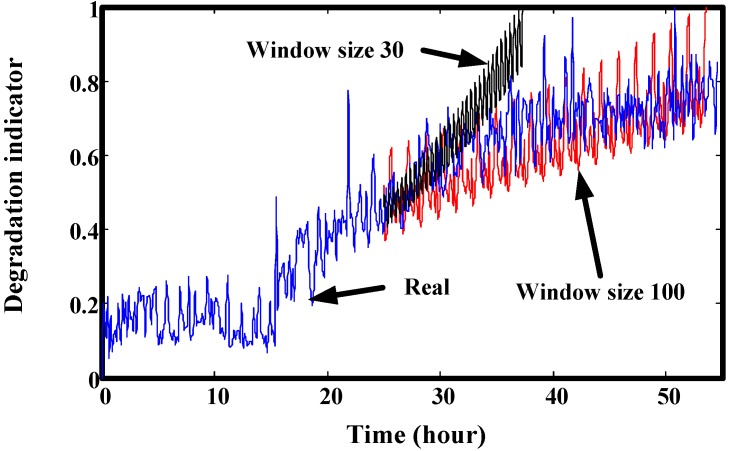
Prediction results comparison using different window sizes (start at 25 h).

As shown in Figure 5, suppose the prediction time is *t*. Then, indicators [*y_i_*_,_ …, *y_o_*, …, *y_t_*_−1_, *y_t_*] are used for training. [*y_i_*, …, *y_o_*_−1_, *y_o_*] are used as input. [*y_o_*_+1_, …, *y_t_*_−1_, *y_t_*] are used as output. For window size, it should not be too big or too small. Too big window size may make the prediction results over dependent on the whole trend of the training data. It means that the predicted indicators may have a similar trend as the training data. However, the real trend of increase of future unknown indicators may be very different from the training data. This may lead to big deviations between the real values and predicted values. This phenomenon is explained by Figure 6. On the contrary, too small a window size may make the overall tendency not be reflected well and lead to problems as follows: descending, increasing dramatically, and stable. The three typical results from small windows are illustrated in Figure 7. The prediction results caused by small windows are explained by Figure 8.

In Figure 6, the real data is from a run-to-failure process of a generator shaft in a helicopter. The data is also used in Section 6 to validate the proposed prediction method. Figure 6 shows that smaller window size has a better prediction effect than bigger window.

Figure 7 shows the three typical results from small windows. One is increasing. In a small local region, the degradation indicators are increasing rapidly, so if they are used to train the prediction model, the predicted values will increase rapidly like the training data. This may lead to a bigger difference from real RUL. Similarly, descending and stable training data may make the predicted values unable to hit the predefined threshold. Figure 8 is an example for the prediction effect with a smaller window.

From the above analysis, we can see that selecting an appropriate window size is important for effective prediction. In order to adapt to indicator variation trends, the window size should be updated in a timely way when new indicators become available. Mean input and output values of window size can be calculated as follow:

(2) $$m_{i}=\frac{\sum_{j=i}^{o}y_{j}}{o-i},i,j,o\in \left[0,T\right],i<j\leq o$$

(3) $$m_{o}=\frac{\sum_{j=o}^{t}y_{j}}{t-o},j,o,t\in \left[0,T\right],o<j\leq t$$

So, an increasing rate can be represented as the ratio of *m_o_* and *m_i_*:

*r* = *m_o_*/*m_i_* (4)

In order to illustrate the increasing degradation as asset usage increases, an adjustment factor *f* is introduced. Its range is (0, 0.1]. According to the test, the appropriate value of *f* is 0.02. If *r* is smaller than 1 + *f*, it means that training data has not obvious increasing trend. Then, the window size should be decreased. The window size shrinking should be stopped when it reaches a predefined threshold. We use *L* to denote the low bound of window size. In the adjustment process, if *r* is bigger than 1 + *f*, the corresponding window size is selected for prediction. Otherwise, the threshold value *L* should be selected as the window size to predict under the condition that *r* is greater than 1.

#### 3.1.2. Window Size Adjusting When It Contains Change Point

**Figure 9:**
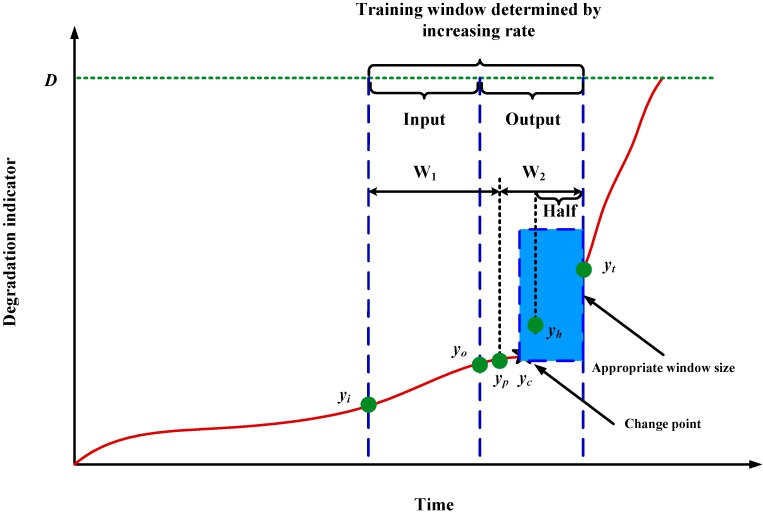
Schematic diagram of window adjusting when change point exists near prediction time *t*.

When an appropriate window size is determined using the method described in Section 3.1.1, it is necessary to check whether this window contains a change point or not. As shown in Figure 9, *y_c_* is a change point. For degradations of mechanical components, they are always undergoing several degradation states which can be reflected by indicators extracted from vibration signals. We regard the points connecting different degradation states as change points. Figure 9 shows how the indicators of the left and right sides of the change point have different increasing trends. If we use the window size determined in Section 3.1.1, there predicted indicators may show a big deviation, so we need to adjust the window size further. Ideally, the window size should be adjusted to the blue region surrounded by the blue dotted line. Namely, indicator vector [*y_c_*, …, *y_t_*] should be used as training data. In order to adjust the window size automatically, a judging rule should be developed.

The window determined by Section 3.1.1 can be continually divided into two parts: W_1_ and W_2_. Then, the indicator vector in window W_1_ is [*y_i_*, …, *y_p_*] and in window W_2_ it is [*y_p+_*_1_, …, *y_t_*]. The following equation is used to judge whether the original window contains a change point or not:

(5) $$\begin{array}{l} a=\text{max}\left[y_{p},\ldots ,y_{t}\right]-\text{min}\left[y_{p},\ldots ,y_{t}\right]\\ b=\text{max}\left[y_{i},\ldots ,y_{p}\right]-\text{min}\left[y_{i},\ldots ,y_{p}\right]\\ a\geq b \end{array}$$

If a change point is detected near the predicted time *t*, then, window W_2_ is used as the new training window. Half indicators are used as input. The remaining indicators are used as output, *i.e*., [*y_p_*, …, *y_t_*] is used as the final prediction window. However, the selection of point *y_p_* which divides the original window is very important. The minimum window length used for training is *L* as mentioned in Section 3.1.1, so the initial value of W_2_ should be selected as *L*. Then, *y_p_* gradually shifts towards the left and its location (right or left of the change point) is determined. The location can be judged by the distance from the point to a straight line fitted by indicators of W_2_. Finally, we can find an appropriate window in which *y_p_* and *y_c_* are overlapped. *y_h_* denotes the middle of window W_2_.

**Figure 10:**
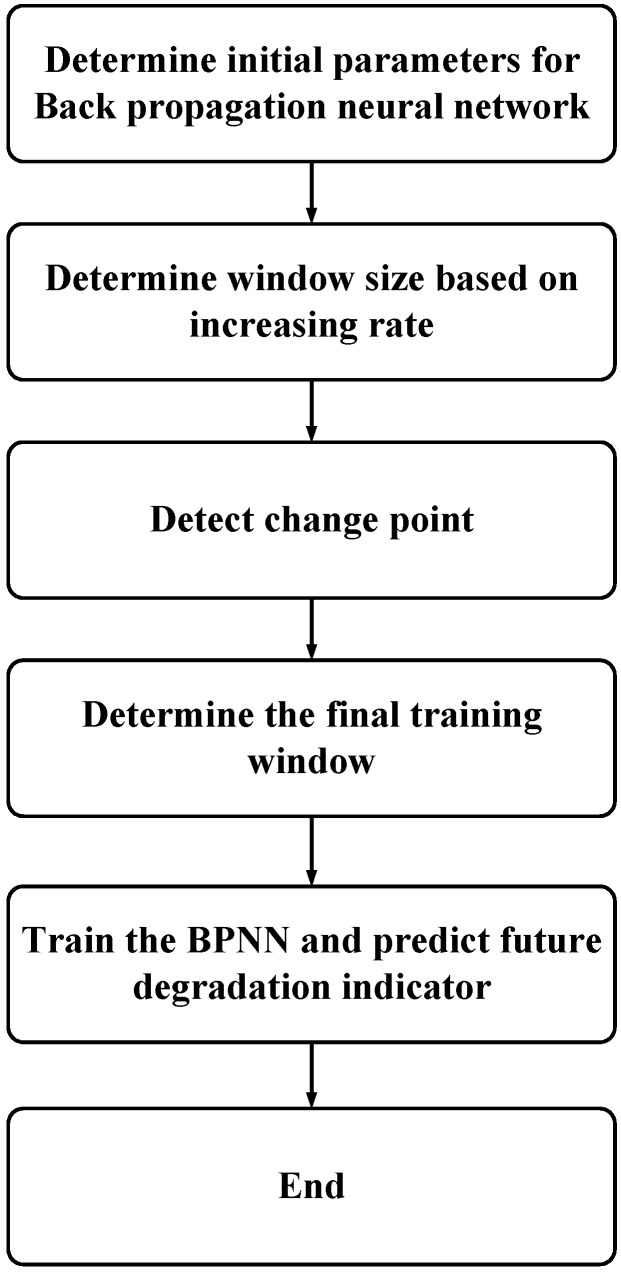
Whole process of RUL prediction based on the proposed method.

### 3.2. Rolling Prediction and Limitation

For the known degradation indicators, if no change point is detected, then the window determined in Section 3.1.1 is used as the training data. Indicator vector [*y_i_*, …, *y_o_*] is the input data. Indicator vector [*y_o+_*_1_, …, *y_t_*] is the output vector. Generally, because of the increasing degradation, predicted values should be greater than associated input values. Meanwhile, predicted values cannot be too big, otherwise, it may lead to great variance between the real and predicted values, so we need to give a limitation for predicted values. Taking training data as an example, *y_t_* should be greater than *y_o_*. Otherwise, these two indicators are at least equal. It can be explained as the following equation:

(6) $$\begin{array}{l} \text{if}\;y_{t-j}<y_{o-j}\\ \text{then},y_{t-j}=y_{o-j},j\in \left(0,\frac{t-i}{2}\right) \end{array}$$

If the predicted value is too big, a limitation is as follows:

(7) $$\begin{array}{l} \text{if}\;y_{t-j}>\left(1+f^{\prime }\right)y_{o-j}\\ \text{then},y_{t-j}=\left(1+f\prime \right)y_{o-j},j\in \left(0,\frac{t-i}{2}\right) \end{array}$$

Then, rolling prediction can be implemented until a predicted value hits the predefined threshold. Finally, RUL can be acquired. Details about window size determination and prediction process are illustrated in Figure 10.

## 4. Validation by Simulated Degradation Data

**Figure 11:**
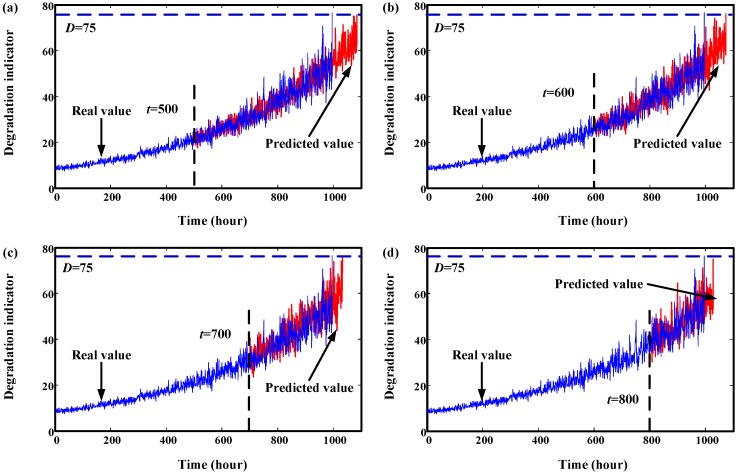
Trend prediction results of simulated data of Component 1. (**a**) prediction results at start time 500 h; (**b**) prediction results at start time 600 h; (**c**) prediction results at start time 700 h; (**d**) prediction results at start time 800 h.

**Figure 12:**
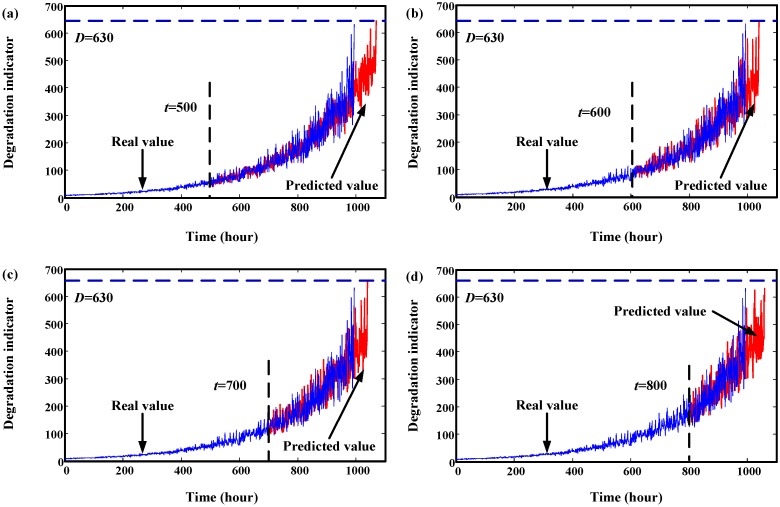
Trend prediction results of simulated data of Component 2. (**a**) prediction results at start time 500 h; (**b**) prediction results at start time 600 h; (**c**) prediction results at start time 700 h; (**d**) prediction results at start time 800 h.

In order to validate the proposed neural network-based prediction method, two degradation datasets are simulated. Details about the simulation process is explained in Section 2.3. These two components are from different machinery systems, so their degradation processes are different. Parameters for neural network can be found in Section 2.3. Initial window size is 300. The minimum window size *L* is 20. Their trend prediction results are illustrated in Figure 11 and Figure 12. From these two figures, we can see that the effects are very good. For Component 1, failure threshold *D* is set to 75. For Component 2, failure threshold *D* is set to 630.

**Figure 13:**
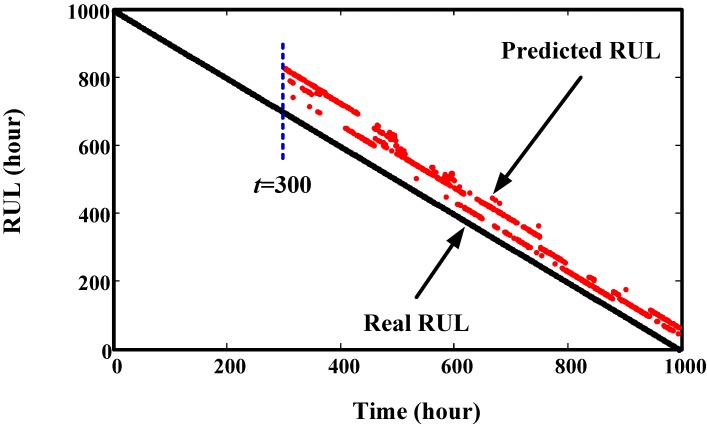
Prediction results of Component 1 at different time points.

**Figure 14:**
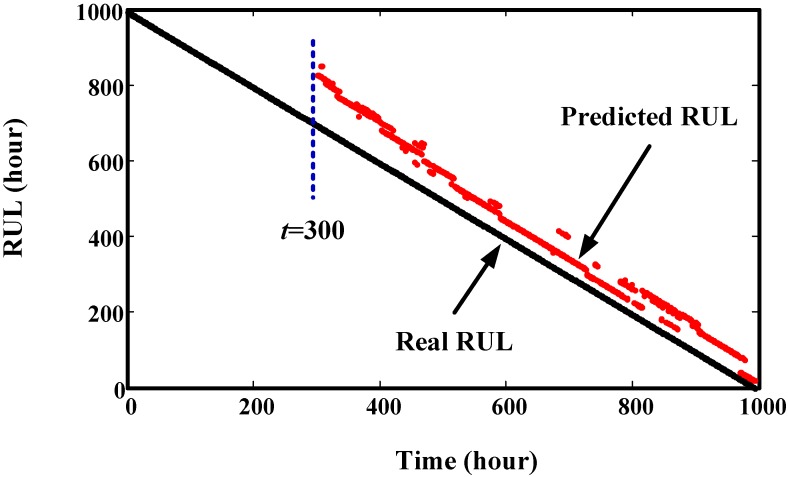
Prediction results of Component 2 at different time points.

The overall prediction effects of Component 1 and Component 2 at different times are given in Figure 13 and Figure 14, respectively. From these two figures, we can see that the predicted RULs are close to the real RUL, demonstrating the effectiveness of the proposed method.

## 5. Validation by Gearbox Run-to-Failure Data

**Figure 15:**
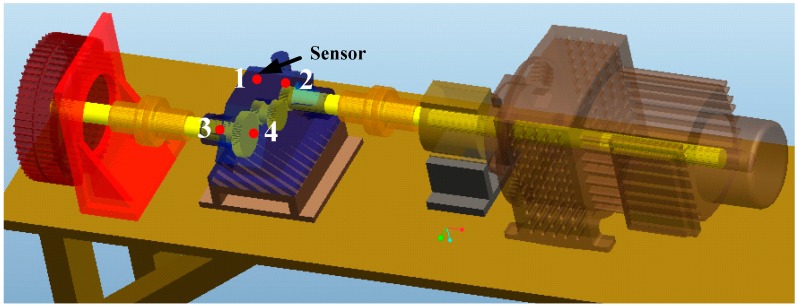
Three dimensional graph of the gearbox test-rig.

Figure 15 is a three-dimensional diagram of an experimental gearbox system. It includes a gearbox, electric motor, and magnetic powder brake. Four accelerometer sensors are mounted on the gearbox above the bearings. The gear on the low speed shaft has 81 teeth and meshes with the gear on the intermediate speed shaft with 18 teeth. The gear on the high speed shaft has 35 teeth and meshes with the gear on the intermediate speed shaft with 64 teeth.

This test is a run-to-failure test, *i.e*., from normal to failure. When the amplitude of the signal exceeds 60 m/s^2^, the experiment is stopped. The whole test took 548 h under the speed of 1200 rpm and 15 Nm load (input side). For every inspection point, the sampling frequency is 20 kHz and lasts 12 s. The interval between two consecutive inspections is ten minutes. After overhaul, it is found that the main fault mode of the gearbox is gear wear. All the teeth of low speed shaft gear are broken, while the other gears only have slight wear. Detailed information about the run-to-failure test can be found in [31].

**Figure 16:**
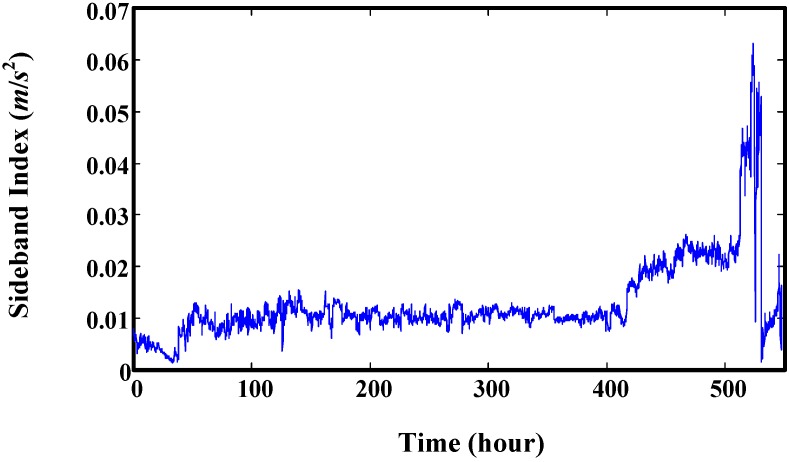
Sideband index developed in [31].

A sideband index developed in [31] is directly used in this paper to validate the proposed prediction method. This sideband index of this gearbox is illustrated in Figure 16.

**Figure 17:**
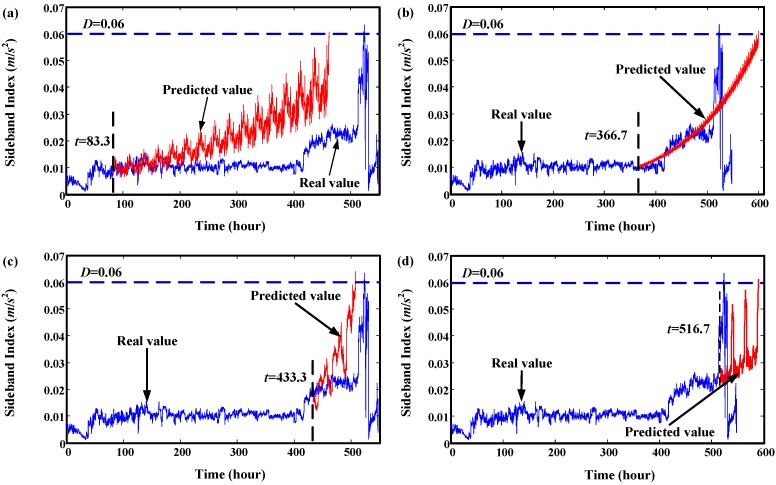
Trend prediction results of degradation data of gearbox. (**a**) prediction results at start time 83.3 h; (**b**) prediction results at start time 366.7 h; (**c**) prediction results at start time 433.3 h; (**d**) prediction results at start time 516.7 h.

**Figure 18:**
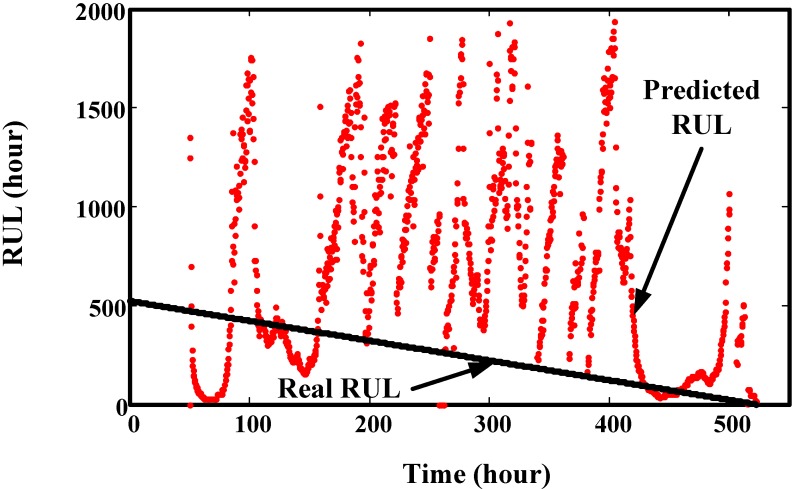
Comparison of real RUL and predicted RUL of the gearbox degradation data.

**Figure 19:**
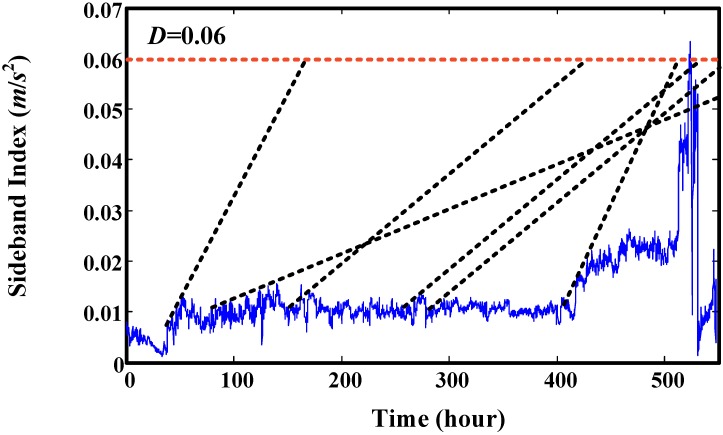
Explaining the great variance of predicted RUL values.

Then, this set of degradation indicators are used to validate the proposed method. We select four time points to explain the prediction effect of proposed model. The results are illustrated in Figure 17, which shows that the predicted values mainly depend on the data used for training. The predicted trend values are very similar to the real trend of the training data. RUL prediction results at every time point are given in Figure 18. It shows that there are big variances between the predicted RUL and real RUL. Most of the predicted RULs are bigger than the real ones. This is because the degradation indicators in the early stage are flat. The trend of predicted values extends to a long distance. For the smaller predicted values, the reason is there are some fast increasing phases. For indicators in these fast increasing phases, the trend of predicted values maybe very similar to their trend. This leads to a smaller RUL value. This can be seen in Figure 19. For this experiment, the initial window size is selected as 300 (time points-10 min a point). The minimum window size *L* is 20.

## 6. Validation by Helicopter Shaft Run-to-Failure Data

**Figure 20:**
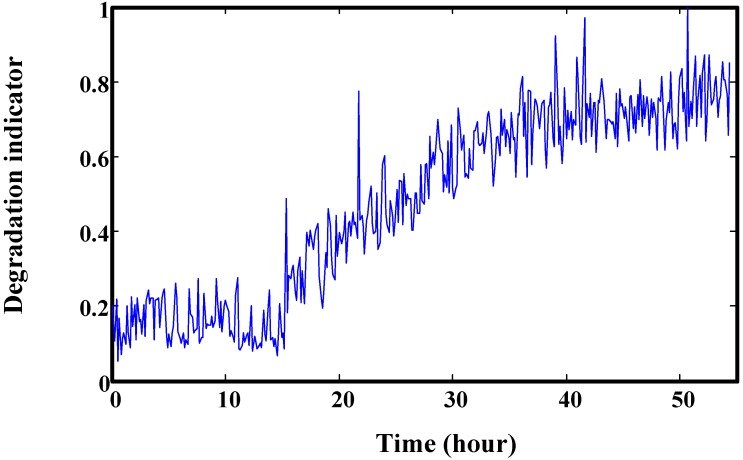
Helicopter normalized degradation indicator of the left shaft of the generator.

This helicopter shaft failure data was acquired by a health and usage monitoring system (HUMS). This shaft is a generator shaft which has the highest wear rate. Its degradation severity can be directly estimated through the amplitude of shaft order 1, 2 or 3 signals in the frequency spectrum. Finally, three shaft orders were mapped into one value in the range [0, 1]. A Rayleigh hypothesis test was used to judge the alarm. Detailed information can be found in [32]. Figure 20 is the degradation indicator of one generator shaft.

**Figure 21:**
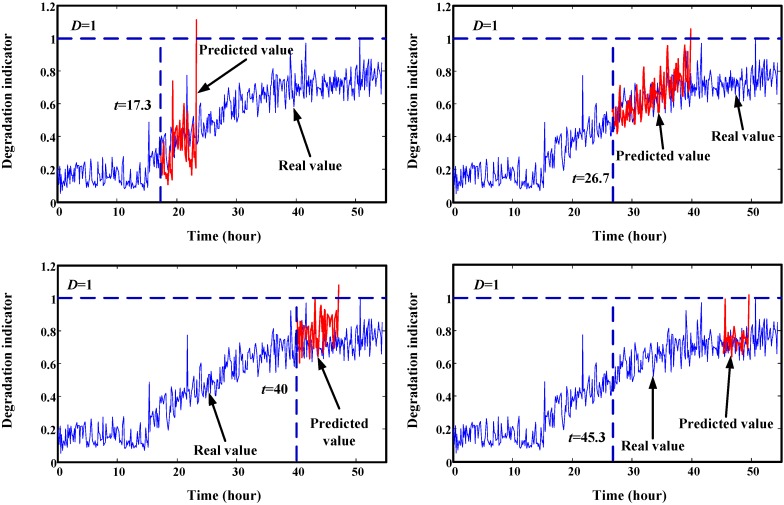
Trend prediction results of degradation data of gearbox. (**a**) prediction results at start time 17.3 h; (**b**) prediction results at start time 26.7 h; (**c**) prediction results at start time 40 h; (**d**) prediction results at start time 45.3 h.

**Figure 22:**
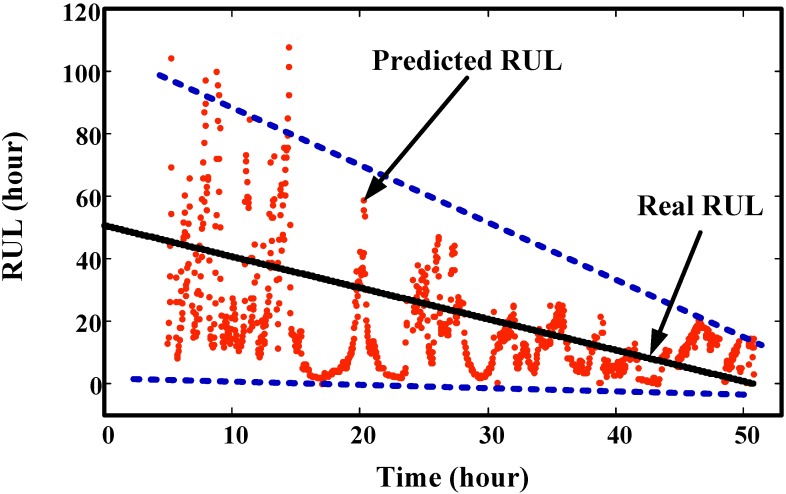
Comparison of predicted RUL values with real RUL values at different inspection time points.

Trend prediction results of the helicopter generator at four time points are illustrated in Figure 21. It shows that the predicted values are overlapped with the real values. This demonstrates the good prediction effect of the proposed method. The RUL prediction result at each inspection time is given in Figure 22. We can see that the variances of predicted RUL are gradually decreasing. Compared to the gearbox RUL prediction, it is more effective for the helicopter degradation prediction. In order to acquire more data, we interpolated values into this degradation trajectory. This does not change the original trend. For this test, initial window size is selected as 100. The minimum window size is 20.

## 7. Conclusions

This paper develops an adaptive trend prediction method. It can adjust window size according to the variation of the degradation path. It overcomes a main problem of many existing prediction methods, namely the poor extrapolability. The proposed method can predict RUL when no failure or suspension histories can be used. It predicts future indicators mainly based on the training data trends, so training data selection is very important. The dynamic window adjusting method developed in this paper is its main contribution. Finally, two simulated sets of degradation data and two real run-to-failure datasets are used to validate the prediction method. The results demonstrate the proposed method can effectively predict RUL. The topic needs however further research for non-stationary variation degradation indicators, it needs to research further.
